# The Motive and Method of Pelvic Surgery

**Published:** 1890-12

**Authors:** Joseph Price

**Affiliations:** Philadelphia


VOL. VII. DECEMBER, 1890. NO. 10.

### Original Communications.

### THE MOTIVE AND METHOD OF PELVIC SURGERY.*

BY JOSEPH PRICE, M. D., PHILADELPHIA.

The peculiar position assumed by the opponents of pelvic surgery, and of those who pretend to obviate its necessity and disprove its justifiability in many cases in which the experienced surgeon can see no other way out of the difficulties he encounters, is sufficient explanation for the presentation of this paper before your Society.

Pelvic surgery must be considered apart from abdominal surgery. It is distinct from it, both in the nature of the lesions dealt with, in the difficulties it presents, and in the complications and embarrassments to routine technique.

A little detailed consideration will suffice to explain this point. The drainage of a large suppurating abdominal cyst through the abdominal wall used to be a common manner of allowing nature to complete an operation with which the surgeon could not suc-

* Read before the Southern Surgical and Gynecological Association, at Atlanta, Nov. 11th, 1890.

cessfully cope by his art. If we look over the records of pelvic surgery of later years, we find that the same method has been tried and recommended in the surgery of pelvic abscesses, etc. Incomplete surgery of this sort, together with its unsuccessful and tedious termination, probably gave rise to the method of attempting to deal with disease of purulent nature in the pelvis by puncture through the vagina. Adhesions that cannot but be barely separated by all the delicately refined force of the skilful finger are the Bluebeard of the unpracticed, and an easier method of dealing with them without touching them is one of the intrenchments of bad work not yet argued out of the field of surgery.

Nowhere as much as in pelvic surgery does the distinction between the general surgeon and the specialist in pelvic disease stand out so clearly. Pelvic adhesion in appendicitis, for instance, Mr. Treves would deal with by the knife. If this is feasible, why not put the knife to ovarian and tubal abscess, to all intestinal fixation by inflammatory process and the like ? The very suggestion of such method to the mind of the specialist, accustomed to deal with all the complexities of pelvic surgery, is fraught with evil, and this mere suggestion only makes it clear that general surgeons, in so far as they are entirely wedded to the knife in removing disease, fall short of the demonstrated harmfulness of its application in pelvic work. A careful observation of the surgery of the grosser abdominal kind serves clearly to show that abdominal surgeons even, especially those of the earlier period, are apt to fall short in the attainments of pelvic surgery, by applying only the methods of abdominal surgery to pelvic disease. The truth of this proposition will become more apparent if it is considered how few, practically, of the elder ovariotomists have been really successful in dealing with pelvic disease. Pelvic surgery in reality is a much later branch of the art, and needs a separate niche in the records of its accomplishments. The fact, then, that a separate division of the surgery of the general abdominal cavity has been created is clearly suggestive evidence that the motives and reasons for such surgery are just as clearly defined.

What is it that has differentiated this division from the surgery of the general abdominal cavity? Clearly, first, its pathology. The gross lesions of the ovarian cyst could easily be distinguished, but how many women perished from the concealed misery of a pelvis bound dermoid! The enormous fibro-cystic tumor of the uterus, in most instances, could be clearly made out, yet what of the innumerable multitudes of those who have fallen victims to hæmatocele, so called, to cellulitis—yet a shroud for mummified imbecility, to pus in the tubes and ovaries, and to peritonitis and childbed fever—all visitations of Providence.

The careful observer of clinical manifestations looked for a new pathology in certain diseases of women characterized by them, and the discovery of this newer pathology gave birth to newer modes of treatment, forever to depose poultices and opium—fatal Circe's cup to the patient, though soothing to surgeon of yore.

From the initiation of this new conception of the cause of some of the general manifestations of disease hitherto obscure, a new rationale of procedure arose, and to this we must now look. First of all, successful pelvic surgery cannot hope, unless exceptionally, to deal with pain alone. Pain is a manifestation too general to be dealt with specifically by surgery, unless in the presence of a well-defined lesion. Hence, just in proportion that an operation is done to relieve a symptom only, just so far will it be likely to be a failure. The exceptions will prove the rule. These are the cases likely to fall into the hands of the electricians, and likely also to return to the hands of the surgeon with a lesion no longer difficult of discovery. The methods of the electrical treatment all tend towards this end. The indiscriminate intra-uterine application, curetting and the like, all stand on the same ground. Further, just as it is undesirable to operate in the absence of a well-defined lesion, so the motives for operation may be clearly set forth by a well-defined series of indications and conditions, all of them founded upon a real pathology. The existence of general pelvic adhesions, by which the intestinal viscera are tied fast to the uterine system, whereby all the functions of both systems are interfered with, constitutes one of the most annoying indications, for the relief of which there is often urgent necessity. Here we are opposed by the "cellulitis" doctrine and the electrical panacea, and taught to "melt" down adhesions like snow before the sun, and other poetic metaphors, useless as music, which nowhere else than in fable can subdue savagery. Nowhere but in fable will adhesions melt away. A number of years ago there appeared in the *Transactions of the American Gynecological Society* a lengthy consideration of alleged cures of ovarian cysts by electricity. Many years before there appeared learned discussions on Perkin's tractors and Berkeley's tar-water. The electricians yet talk learnedly of the undetermined place of electricity in the treatment of ovarian cysts, but tar-water and tractors have gone to their long rest. The time must yet come when the claims made for electricity as an universal panacea must be exploded, and its real, limited and narrow horizon of usefulness be well defined. The pernicious effect of so-called cures of reported complicated cases, adhesions, inflammations and the like, by men without training, who look only at the Ampèremeter while they adjust a clay pad or introduce a galvanic sound, is not to be overestimated. I have repeatedly shown, by exhibited specimens, the fallacy of the claim of exact diagnosis by these men and the arguments are irrefutable. Claims are nothing when refuted by facts. I believe that the only position assumed by the electricians that has the slightest foundation in fact, is that electricity will sometimes control hemorrhage and relieve pain. That it cures either is not proven. I expect that this expression of opinion in regard to electricity will be construed as an admission of the mode of treatment.

Now, apart from general adhesions referred to above, there is a class of diseases, separate in itself and often indistinguishable as a gross lesion, which must be considered, though territorially small. I refer to fimbrial occlusion. Here is a condition which often, in protracted suffering, not yielding to well-directed, non-meddlesome treatment, exploratory incision may often reveal. It is obvious that all the electricity of all the dynamos and batteries in existence cannot break up and negative the pathological effects of such lesions as these here shown. You will see that the adhesions are as strong as the tube itself; nay further, there is often an inflammatory constriction of the intestine, as strong as the intestinal wall itself, which we may as soon expect to melt away as the adhesive, constricting bands.

We have in this condition a corresponding one of complete sterility, lancinating pain and general discomfort, and where must we find relief unless in the removal of the offending lesion. Electricity, we are encouraged to believe, will make each fimbrial adhesion to soften as wax before its genial flow, free each part and fibre of adherent fimbriæ, and envelop the egg-producing ovary with the nursing fold of a restored fimbria. I beg you all examine these specimens and honestly decide whether it is not all a delusion and a lie.

Not long since I received a letter from a well-known advocate of electricity and conservatism, flattering in it approval of my results and coinciding, for the most part, with all my expressed opinions concerning the treatment of diseases of the kind here discussed, but begging a place for electricity, because there must be a place for those men who cannot get results by surgery. Is such a position fair, is it honest, is it scientific? The argument is all the stronger, recollect, because the name of this man is prominently quoted in all electrical discussions, as a writer of a book and as an authority.

The newer pelvic surgery attempts to relieve chronic displacements of the uterus by gentle means, not forcible. It recognizes the danger of forcible interference with the sound, and when it reads the records of violent inflammations set up, of death by hemorrhage from such interference, it agrees that it is a condition, not a theory, that confronts us, and rather resorts to direct surgery to restore a displaced uterus than to force out the adhesions by breaking what it cannot afterward control. The lessons learned by surgery of this sort teach us that uterine displacements not only involve the uterus, but are complicated by adhesions of every variety, tubal, ovarian, appendical, intestinal, and that these cannot be carelessly or forcibly dealt with, unless they are put directly under the eye of the operator, and their complications thereby under control. The modern latter-day pelvic surgery recognizes the undiscovered frequeney of extra-uterine pregnancy. It sees in it a lesion deadly from its inception until its end. It recognizes and proves, not theoretically but practically, that all its multitudinous complications can be dealt with in no other way than surgically, and has fought its way up to what must be the universal abandonment of all other theoretic modes of treatment.

It shows the vicious position to all progress and real surgery, assumed by pseudo-surgeons who pose as innovators, without knowledge first or second-hand, who prate learnedly of morality and infanticide, who urge the claims of an impossible fœtus against the life of the mother of the family. For a monumental record of ignorance such as this there is no excuse. Its effects are more vicious in surgery than is *Kreutzer Sonata* to family morals. Reputable journalism should banish such sensation-mongers from its columns. I might thus continue along the various paths of pelvic surgery, showing at each step that the motives of its art are preservative, logical and honest; that it deals with disease as it is found, not as it is imagined; that it takes the short road and the least painful to establish a cure; that it would remove an offending, useless body or organ rather than tolerate it as a perpetual menace to the remaining economy. With such an aim, its utility cannot be questioned; its honesty is determined; its permanency assured.

Let us consider now for a little its methods. These have for their insignia, directness, simplicity, regularity, varied according to the variations of individual cases. It frees all operations from the nature of a mere exhibition, and while it would admit observers, it does so to teach, not to hippodrome. It places each observer at the vantage-point of an intellectual spy, and gives him an insight into both the abuses of useless surgery and the pretenses of palliation.

The steps of each operation after incision can only be determined by exploration. Primarily in pelvic disease adhesions are likely first to demand attention. These may be localized or general. It is first to be remembered that there are some cases so complicated by adhesions that they must be abandoned as exploratory only. These are in the main, I believe, malignant. The adhesions are apt to be rendered distinctly worse by prolonged electrical treatment. This has been observed in many of my own cases. This point should be a matter of direct investigation in every case that comes to us for operation.

In dealing with adhesions the first point to be sought after is to find a crease or crevice into which some progress can be made. This is a matter often of the keenest difficulty; many cases which at first appear utterly unassailable will, by perseverance, yield to well-directed effort, and from that time on the enucleation of the mass be comparatively easy. It is to be remembered that violence is not to be attempted. Sufficient force to accomplish the separation of adhesions, if it be violent, cannot be other than harmful. I cannot better express it or explain it than by saying that the force should be as a gentle momentum, not as a velocity and momentum. In separating intestinal adhesions they should be broken as far from the bowel as possible. The farther away, the less liable will they be to bleed, and the absence of hemorrhage is a great comfort in these cases. The strings of adhesions may be dealt with according to their size, it sometimes being best to remove them; at others there is no necessity for this. In doubtful cases their removal is the better surgery. All bowel adhesions should be carefully examined after their separation. By so doing, fæcal fistulæ will often be avoided by the careful placing of the intestinal suture. It hence is apparent that no pelvic surgery should be attempted until the operator is competent to deal with intestinal wounds even to resection and anastomosis. Once the adherent mass is removed the ligature should be applied close up to the *cornu uteri*. The ligature should not be so heavy as to resist knotting, nor so light as to break easily. The ordinary surgical knot is the safest of all knots with which to tie the pedicle. It constricts more evenly and certainly, and will slip less readily. The leaving of sufficient button is of the greatest importance to prevent slipping of the ligature. In dealing with all abscesses, cavities or pus in any shape, all débris should be cleaned out by scraping, if necessary, and careful drenching of the pelvis and its recesses practiced. The procedure itself will be a comfort in doubtful cases, since it is the best of all methods with which to relieve shock, and cannot possibly do harm. There is nothing doubtful or theoretical in this advice. I use it constantly in cases so ill that the ether is taken away almost as soon as the incision is made, and its effect is, well—marvellous. In the use of drainage it is best to err on the safe side, and I prefer to place a drainage in a doubtful case. The tube I prefer I here exhibit.

In the treatment of extra-uterine pregnancy my urgent advice is to operate without delay when the symptoms point to the disease, with the assurance that delay will only complicate matters and sacrifice the life of the mother. I have now so often proven the correctness of this position, both by my own cases and those of others, that I shall not dwell on it here, but illustrate it further in the discussion. Drainage, in these cases, is to be followed out as a routine procedure, unless in unruptured tubal pregnancy, which, if it is found, is a matter of congratulation both for mother, and surgeon, and family. In the treatment of appendicitis I urge you to accept the teaching of pelvic surgery, as reinforced by scientific pathology. Remember that this lesion is to be considered as the real presence of a foreign body, and that its removal is just as much called for as is that of a calculus from the urinary or gall-bladder. That there are perityphlitic abscesses is no argument against the fact that they are rare and the exception. These cases are often to be treated, on account of delay, by simple incision and drainage, but where it is possible the appendix is to be removed.

I have thus endeavored briefly to bring before your Society the salient points of pelvic surgery in the light of its most recent successes and conflicts. I have endeavored to show that it's field is not one of experiment or palliation; that it strives in all cases to remove the offending body in order to conserve the rest of the economy; that its tenets are founded on philosophy and fact, not fiction, and that its worth lies in its proven results.

It has reached a standard not to be measured by tyros and dabblers, without instinct of the art, and without energy to train for that of all things so difficult to attain, the conception of the limitation of all art so as to comply with the limitations of nature and natural laws. The surgery that plucks out the eye or casts aside the limb to save the eye, or the limb, or the life, is greater, better and wiser than a sentiment that preserves a shell to inclose a ruin.

